# Changes in electroencephalographic power and bicoherence spectra according to depth of dexmedetomidine sedation in patients undergoing spinal anesthesia

**DOI:** 10.7150/ijms.54677

**Published:** 2021-03-15

**Authors:** Kwon Hui Seo, Kiseong Kim, Soo Kyung Lee, Jeonghoon Cho, Ji Hyung Hong

**Affiliations:** 1 Department of Anesthesiology and Pain Medicine, Hallym University Sacred Heart Hospital, College of Medicine, Hallym University, Anyang, Republic of Korea.; 2 Strategic R&D Center, Biobrain Inc. 723, 408 Daedeok-daero Seo-gu, Daejeon City, Republic of Korea.; 3 Division of Oncology, Department of Internal Medicine, Eunpyeong St. Mary's Hospital, The Catholic University of Korea, Seoul, Republic of Korea.

**Keywords:** bicoherence, bispectral index, depth of anesthesia, dexmedetomidine, power spectral analysis, quadratic phase coupling

## Abstract

**Background:** Assessment the depth of dexmedetomidine sedation using electroencephalographic (EEG) features can improve the quality of procedural sedation. Previous volunteer studies of dexmedetomidine-induced EEG changes need to be validated, and changes in bicoherence spectra during dexmedetomidine sedation has not been revealed yet. We aimed to investigate the dexmedetomidine-induced EEG change using power spectral and bicoherence analyses in the clinical setting.

**Patients and Methods:** Thirty-six patients undergoing orthopedic surgery under spinal anesthesia were enrolled in this study. Dexmedetomidine sedation was conducted by the stepwise increase in target effect site concentration (Ce) while assessing sedation levels. Bispectral index (BIS) and frontal electroencephalography were recorded continuously, and the performance of BIS and changes in power and bicoherence spectra were analyzed with the data from the F3 electrode.

**Results:** The prediction probability values for detecting different sedation levels were 0.847, 0.841, and 0.844 in BIS, 95% spectral edge frequency, and dexmedetomidine Ce, respectively. As the depth of sedation increased, δ power increased, but high β and γ power decreased significantly (*P* <0.001). α and spindle power increased significantly under light and moderate sedation (*P* <0.001 in light vs baseline and deep sedation; *P* = 0.002 and *P* <0.001 in moderate sedation vs baseline and deep sedation, respectively). The bicoherence peaks of the δ and α-spindle regions along the diagonal line of the bicoherence matrix emerged during moderate and deep sedation. Peak bicoherence in the δ area showed sedation-dependent increases (29.93%±7.38%, 36.72%±9.70%, 44.88%±12.90%; light, moderate, and deep sedation; *P* = 0.008 and *P* <0.001 in light sedation vs moderate and deep sedation, respectively; *P* = 0.007 in moderate sedation vs deep sedation), whereas peak bicoherence in the α-spindle area did not change (22.92%±4.90%, 24.72%±4.96%, and 26.96%±8.42%, respectively; *P*=0.053).

**Conclusions:** The increase of δ power and the decrease of high-frequency power were associated with the gradual deepening of dexmedetomidine sedation. The δ bicoherence peak increased with increasing sedation level and can serve as an indicator reflecting dexmedetomidine sedation levels.

## Introduction

Dexmedetomidine, an α_2_-agonist, causes less respiratory depression and hemodynamic instability than other sedatives 1, and is preferably used as a sedative in patients undergoing regional anesthesia. During spinal anesthesia, high sensory block levels and deep sedation are often required. Sedation scales are commonly used in clinical settings; however, they offer an intermittent and subjective assessment, and can interfere with sedation. In particular, dexmedetomidine produces arousable sedation, which can be awakened even with light stimulation 1,2. Compared to the sedation scales, depth of anesthesia (DOA) monitoring using processed electroencephalogram (EEG) potentially provides continuous, objective assessments. However, most EEG-based DOA monitors do not consider anesthetic agents, which result in high interindividual and inter-agent variability 3, 4. DOA monitors evaluate changes in parameters from processed EEG signals but fail to assess for patterns or features uniquely associated with a specific level of sedation or anesthesia. Depending on the anesthetics, the calculated index can be significantly different at the same depth of anesthesia 4. Investigation of anesthetic agent-induced EEG changes can be helpful in interpreting the calculated DOA monitor value and assessing sedation levels without disturbing sedation. Previous studies have demonstrated that dexmedetomidine-induced sedation shows EEG properties similar to those of non-rapid eye movement (NREM) sleep stages 2-3 5-7. Most of them investigated dexmedetomidine-induced EEG changes using power spectral or coherence analysis during moderate to deep sedation in volunteers. They have been limited by the lack of validation in the clinical setting. In addition, bispectral index (BIS) is the most commonly used DOA monitor, but only a few studies have analyzed the performance of BIS for dexmedetomidine sedation in patients undergoing regional anesthesia.

Bicoherence analysis is a power-independent bispectral analysis using a single EEG channel, which has been developed to detect cross-frequency quadratic phase coupling 8. It allows the investigation of changes in the reentry system such as a reverberating source in the thalamocortical network 9. When a certain rhythm containing both original and reverberating modulated subcomponent is dominantly generated, a phase coupled peak will appear as high bicoherence 8. Therefore, bicoherence analysis can explore the neural oscillations based on reverberating thalamocortical behavior, which is not revealed by power spectral or coherence analysis 9-11. Changes in bicoherence according to anesthetic depth are well known in inhalation anesthetics and propofol 9, 12. However, how it appears in dexmedetomidine sedation has not been revealed yet. Since dexmedetomidine has a different mechanism from that of anesthetics acting on γ-aminobutyric acid (GABA) receptors, the changes in bicoherence spectra induced by dexmedetomidine can be different and this information can contribute to anesthetic agent-specified DOA monitoring.

Therefore, this study aimed to investigate dexmedetomidine-induced EEG changes using a single frontal electrode according to the escalation of sedation level with bicoherence analysis in addition to power spectral analysis in patients undergoing spinal anesthesia. We also aimed to evaluate the performance of BIS and 95% spectral edge frequency (SEF95) to assess the depth of dexmedetomidine sedation.

## Materials and Methods

This study was approved by the Institutional Review Board of Hallym University Sacred Heart Hospital (HALLYM 2019-11-031-002) and registered on the Clinical Research Information Service of the Korea National Institute of Health (CRIS, http://cris.nih.go.kr, identification number: KCT0004739). Written informed consent was obtained from all patients before the study.

### Study protocol

Thirty-six patients aged between 19-70 years with an American Society of Anesthesiologists (ASA) physical status of I to II who were scheduled for elective orthopedic surgery in the supine position under spinal anesthesia, with an estimated operation lasting 1 to 3 hours, enrolled in this study. The exclusion criteria were as follows: uncontrolled obstructive lung disease, diagnosed cerebrovascular disease, medication affecting the central nervous system, history of allergic reaction to dexmedetomidine or bupivacaine, body mass index >30 kg/m^2^, and women who were pregnant or nursing. Patients who could not reach deep sedation (MOAA/S ≤2) and were administered other sedatives were withdrawn.

None of the patients received premedication. Upon arrival at the operating room, the patient's electrocardiogram, peripheral oxygen saturation (SpO_2_), and noninvasive arterial pressure were monitored. BIS values were measured continuously using BIS VISTA™ version 3.0 (Aspect Medical System, Inc., Norwood, MA, USA), a BIS Quatro (Medtronic, Minneapolis, MN, USA) four-electrode sensor was located approximately at positions F3 and FT9 with the ground electrode at Fpz, and the reference electrode next to F3.

After hydration with 6 mL/kg lactated Ringer's solution, spinal anesthesia was initiated with each patient in the lateral decubitus position at the level of the L3-4 or L4-5 interspace using a 25-gauge spinal needle. A 0.5% hyperbaric bupivacaine (bupivacaine hydrochloride [HCl] 5.28 mg/mL; Reyon bupivacaine dextrose HCl injection 0.5%; Reyon) was administered after identification of the intrathecal space. The bupivacaine dose was based on the usual clinical practice guidelines at our hospital. After spinal anesthesia was performed, the patient was switched to a supine position. A pinprick test was performed to test sensation at 3-min intervals to check the sensory block level. Fifteen minutes after bupivacaine injection, the upper sensory block level was confirmed by a stable block level after three consecutive tests.

After confirmation of the sensory block level, sedation was induced with target-controlled infusion (TCI) of dexmedetomidine (200 μg/50 mL; precede premix inj. Pfizer Pharmaceuticals Korea Ltd., Seoul, Korea) using a syringe pump (Pion pump, Bionet Co., Ltd., Seoul, Korea) controlled using Asan pump software (version 2.1.5, http://www.fit4nm.org; last accessed: June 2014) programmed with the pharmacokinetic model reported by Hannivoort et al. 13 expanded by an equilibration rate constant (ke0) for the effect site of the Modified Observer's Assessment of Alertness/Sedation (MOAA/S) scale estimated in the pharmacodynamic model by Colin et al. 14.

The dexmedetomidine effect site concentration (Ce) was predicted by the TCI pump and was initially targeted at 0.8 ng/mL. After reaching targeted Ce, the level of sedation was checked using the MOAA/S scale (0 = does not respond to deep stimulus; 1 = does not respond to mild prodding or shaking; 2 = responds only after mild prodding or shaking; 3 = responds only after name is called loudly or repeatedly; 4 = lethargic response to name spoken in normal tone; and 5 = responds readily to name spoken in normal tone). After checking the MOAA/S scale, the target dexmedetomidine Ce was increased gradually at a rate of 0.2 ng/mL until the MOAA/S scale reached 1. Before each increase in target Ce, sedation level was checked. The sedation level was checked with at least a > 15 min interval. If the predicted Ce did not reach the target Ce within 30 min from the previous MOAA/S scale check, the MOAA/S level was assessed. A single investigator checked the MOAA/S scale in all patients. After the sedation level reached MOAA/S scale 1, the study was completed and the target dexmedetomidine Ce was decreased to maintain adequate sedation (MOAAS 3-4).

The sedation was conducted in preparation for surgery and during surgery. All patients were administered 5 L/min oxygen via facial masks, and end-tidal CO_2_ and respiratory rate (RR) were monitored from the start to the end of sedation. Hypotension (mean arterial pressure <60 mmHg or systolic arterial pressure <90 mmHg) or hypertension (systolic arterial pressure >160 mmHg) were treated with 10 mg of intravenous ephedrine or 0.5 mg of intravenous nicardipine. The occurrence of bradycardia (heart rate <45/min) was checked and treated with 0.5 mg atropine. We also recorded the occurrence of hypoventilation (RR <10 breaths/min) or hypoxia (SpO_2_ <93%).

### Data collection

All monitored parameters (SEF95, electromyogram activity, signal quality index [SQI], and BIS), and raw waveform from the BIS monitor were recorded electronically using vital sign recorder software (VitalDB, https://vitaldb.net) 15 from the time of monitoring application to the end of the surgery. All parameters were saved in Excel (Microsoft Corporation, Redmond, WA, USA) files at 4-s intervals for statistical analysis. BIS and SEF95 with SQI >90 were included in the analysis.

EEG data were recorded at a sampling rate of 128 Hz, and EEG data from the F3 electrode was used for analysis. Data of EEG signal were filtered with a butterworth bandpass filter (-3dB at 0.5Hz and 50Hz) for further frequency-domain analysis in offline processing. To minimize the effect of the noise and the artifact, the data were analyzed after excluding the noisy and artifactual segments through the exclusion criteria (over ± 100 μV) of the amplitude of the signal. First, 2-min EEG segments were selected from all subjects during the awake, eyes closed baseline state. For assessment of dexmedetomidine induced EEG change, the time points that MOAA/S 4,3,2 and 1 was achieved were recorded and the EEG epoch for each MOAA/S scale was selected as 2 min before the MOAA/S scale assessment time.

### Spectral analysis and EEG band power

To compute the power spectrogram, we obtained individual single-channel EEG signals acquired from BIS™. EEG signals were divided into four states during dexmedetomidine sedation: awake state (MOAA/S = 5), light sedation (MOAA/S = 4), moderate sedation (MOAA/S = 3), and deep sedation (MOAA/S = 1-2). Continuous EEG from each sedation level was segmented from raw EEG, and the power spectrum was calculated from the preceding EEG over each sedation level using Mathematica^®^ software (version 12.1, The Wolfram, Champaign, IL, USA). We calculated the multitaper power spectral density (MPSD) using 4-s EEG segments to quantify the frequency power ratio for a given sedation level. We set the following parameters: window length (4 s), overlap (75%, 3 s), time-half bandwidth product (3), and spectral resolution (1.5 Hz). The relative power ratio in each frequency range was calculated in δ (0.5-4 Hz), θ (4-8 Hz), and α (8-12 Hz), spindle (12-15 Hz), high β (15-25 Hz) and γ (25-40 Hz) regions, as the sum of relative power ratio values in each frequency range. The group-level mean relative power was calculated by averaging each power bin over all patients. The frequency and absolute frequency power (10×log_10_μV^2^ [dB]) were plotted on the x-axis and y-axis, respectively.

### Calculation of the relative EEG band power

We computed the relative powers of each frequency region (δ, θ, α, spindle, high β, and γ) using the following equation for baseline and three sedation levels in all patients:


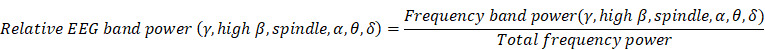


The total frequency power is the sum of δ, θ, α, spindle, high β, and γ band power (0.5-40 Hz). The absolute values of the EEG-derived band power in each patient were calculated, and the comparison of each frequency band power change according to sedation level was analyzed using the ratio to reduce the effect of differences between patients.

### Bicoherence analysis

Bicoherence values were defined as normalized values (range of 0%-100%) and were calculated based on previous studies 9, 16. Bicoherence was computed from two consecutive minutes of artefact-free signals, in all pairs of frequencies between 0.5 and 22 Hz at 0.25-Hz intervals and represented as a two-dimensional density plot. Briefly, the 2-min-long EEG signals from the frontal channel were divided into a series of 4-s epochs, with each epoch overlapping by 75%, resulting in 120 epochs. We applied the multi-taper method to each epoch to estimate the bicoherence. For the Fourier transformation, we applied the Slepian tapers to each epoch 17. Raw bicoherence *BIC (f1, f2)* values were calculated for all pairs of frequencies between 0.5 and 22 Hz at 0.25-Hz intervals, using the following equations 8:


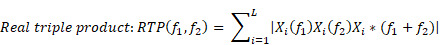



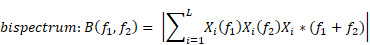



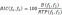


where *i* is the epoch number, *Xi(f_1_)* represents a complex value calculated by Fourier transformation of the *i*th epoch, and X_i_*(f_1_) is the conjugate of *X_i_(f_1_)*. L indicates the number of epochs. We collected the ensembles of the bicoherence spectra of all cases, and each ensemble was averaged under three levels of sedation. Bicoherence is shown as a two-dimensional density plot with x- and y-axis for two coupling frequencies and z-axis for bicoherence. The bicoherence spectra were then presented along the diagonal lines (same pairs of frequencies; f1=f2), calculated with three points moving average, as shown in Fig. 3, to examine the quadratic reentrant behavior of each target frequency rhythm.

The bicoherence and power spectrum showed two peaks in the δ area (0.5-4 Hz) and α to spindle area (9-15 Hz) along the diagonal line of the bicoherence matrix in moderate and deep sedation. We labeled the maximum value of bicoherence in the diagonal lines between 0.5 and 4 Hz as the bicoherence δ peak, and the maximum value of bicoherence in the diagonal lines between 9 and 15 Hz as the bicoherence spindle peak. Next, the mean of each bicoherence peak during the three sedation levels area was calculated to examine the sedation-dependent peak bicoherence changes. Computations were performed using Mathematica^®^ software.

### Statistical analysis

All statistical analyses were performed using SPSS software, version 26.0 (SPSS Inc., Chicago, IL, USA) for Windows (Microsoft Corporation, Redmond, WA, USA). We analyzed the correlation between the MOAA/S scale and BIS or SEF95 or dexmedetomidine Ce using Pearson's correlation analyses. Prediction probability (*P_K_*) values were calculated using Kim's d cross-tabulation statistics 18: *P_K_* = 1 indicates that the measure predicts the observed depth of sedation perfectly; *P_K_* = 0.5 indicates that the predictive accuracy of the measure is no better than chance (50 : 50); and *P_K_* = 0 indicates that the measure has no predictive value. We set the MOAA/S score as the dependent variable and BIS or SEF95 or dexmedetomidine Ce values as independent variables for the Somers' d statistic. One-way analysis of variance (ANOVA) with repeated measures was performed to compare the relative power of six frequency bands and peak bicoherences during different sedation levels. Differences between sedation levels were calculated using a Bonferroni or Dunnett's post hoc test (adjusted *P* value for significance *P* < 0.0083 and 0.0167 in relative band power and peak bicoherence, respectively). The correlation between peak bicoherence and depth of sedation was analyzed using a Spearman rank correlation analysis. *P* values < 0.05 were considered statistically significant for ANOVA and correlation analysis.

## Results

### Patients' characteristics and changes in the BIS value

From February 2020 to July 2020, 36 patients were scheduled to undergo an elective orthopedic surgery under spinal anesthesia were assessed for eligibility, and six patients were excluded because the sedation level did not reach the MOAA/S scale 2 due to short operation time. Therefore, 30 patients were included in the analysis. The demographic and perioperative data of the patients are shown in Table 1. None of the patients showed signs of hypoxia or hypoventilation.

A total of 17 patients reached the MOAA/S 1. Table 2 shows the changes in BIS values, SEF95, and dexmedetomidine Ce according to the sedation level. As the sedation level increased, the BIS value and SEF95 decreased, and dexmedetomidine Ce increased gradually. The *P_K_* values of BIS, SEF95, and dexmedetomidine Ce according to the change in MOAA/S were comparable. BIS and SEF95 were all inversely correlated to the sedation level, and dexmedetomidine Ce was positively correlated with the sedation level (all *P* < 0.001). The Pearson's coefficient of correlation (*r^2^*) was -0.887, -0.843, and 0.896, respectively. When the effect of MOAA/S was controlled, the partial correlation coefficient between SEF95 and BIS was 0.591, which was statistically significant (*P* < 0.001). The coefficient of variation of both BIS and SEF95 was highest in MOAA/S 3 among sedation levels.

### Power and bicoherence spectrum changes

**Figure 1:**
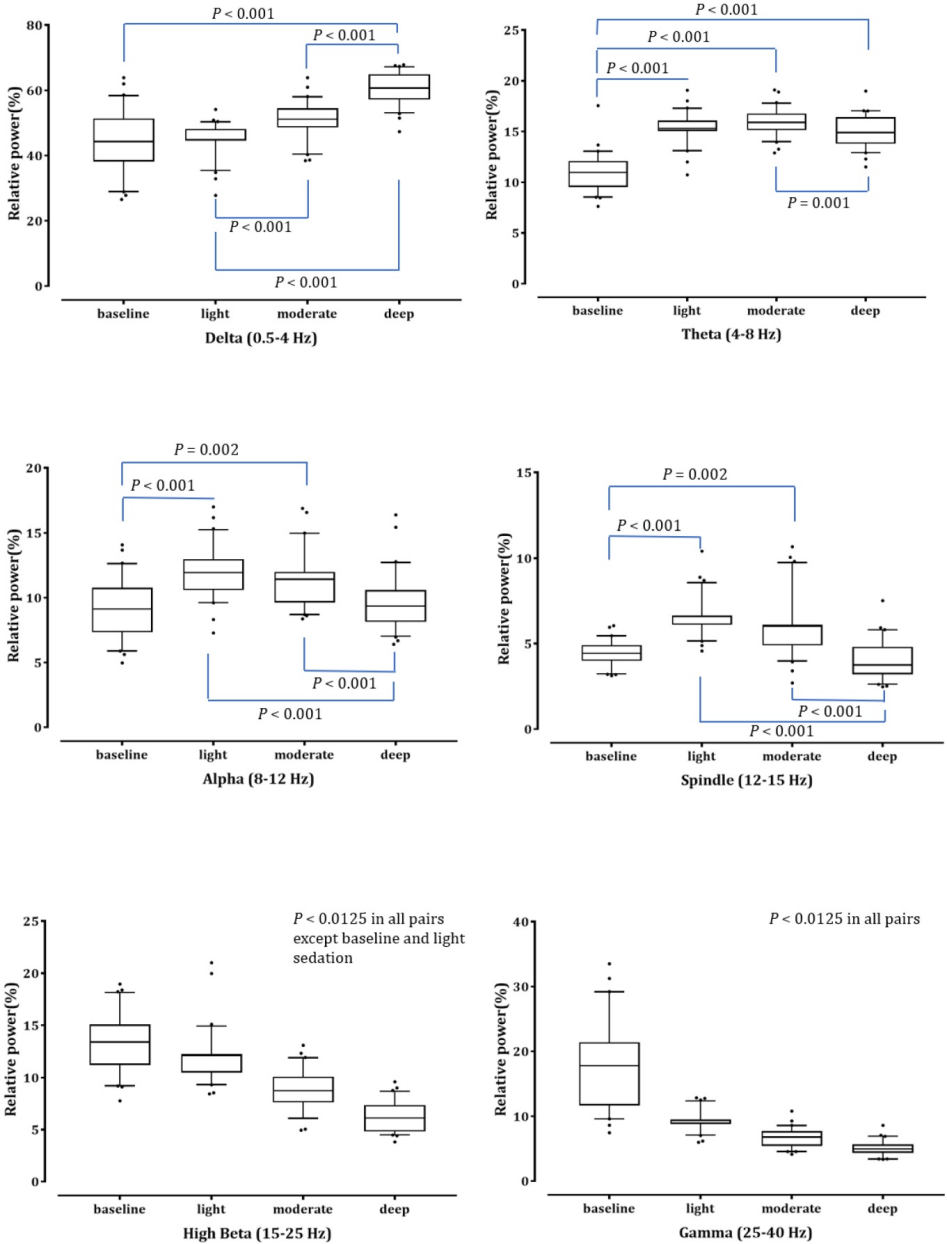
The change in the electroencephalogram (EEG) relative band powers during dexmedetomidine sedation. The box and whiskers plots demonstrate the ratio of EEG band power (δ, θ, α, spindle, high β and γ) at baseline and predefined sedation levels. The upper and lower limits of the box reflect the 75^th^ and 25^th^ percentiles of the sample, and the horizontal line inside each box indicates the median. The upper and lower notches reflect the 90^th^ and 10^th^ percentiles of the sample. Values below and above the notches are drawn as individual points. One-way ANOVA with repeated measures, followed by post hoc Bonferroni or Dunnet test (corrected *P* < 0.0083).

The absolute total power (10
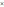
log_10_*μV*^2^) at baseline, light, moderate and deep sedation were 18.42 ± 0.88 dB, 17.75 ± 0.41 dB, 17.62 ± 0.44 dB, and 17.55 ± 0.49 dB, respectively. We compared the relative power of the six frequency bands in the baseline and three-stage sedation levels (Fig. 1). The δ power significantly increased as the depth of sedation increased (*P* < 0.001 in light sedation vs moderate and deep sedation, moderate sedation vs deep sedation and deep sedation vs baseline). The θ power during all sedation levels was significantly higher than that at baseline, and that in moderate sedation was significantly higher than that in deep sedation (*P* < 0.001 in baseline vs light, moderate and deep sedation; *P* = 0.001 in moderate sedation vs deep sedation). α and spindle power changed similarly; they increased significantly under light and moderate sedation but decreased significantly at deep sedation levels (*P* <0.001 in light sedation vs baseline and deep sedation; *P* = 0.002 and *P* < 0.001 in moderate sedation vs baseline and deep sedation, respectively). In contrast to the δ power, high β and γ power gradually decreased significantly as sedation deepened (*P* < 0.001 except baseline vs light sedation in high β power [*P* = 0.42]).

**Figure 2:**
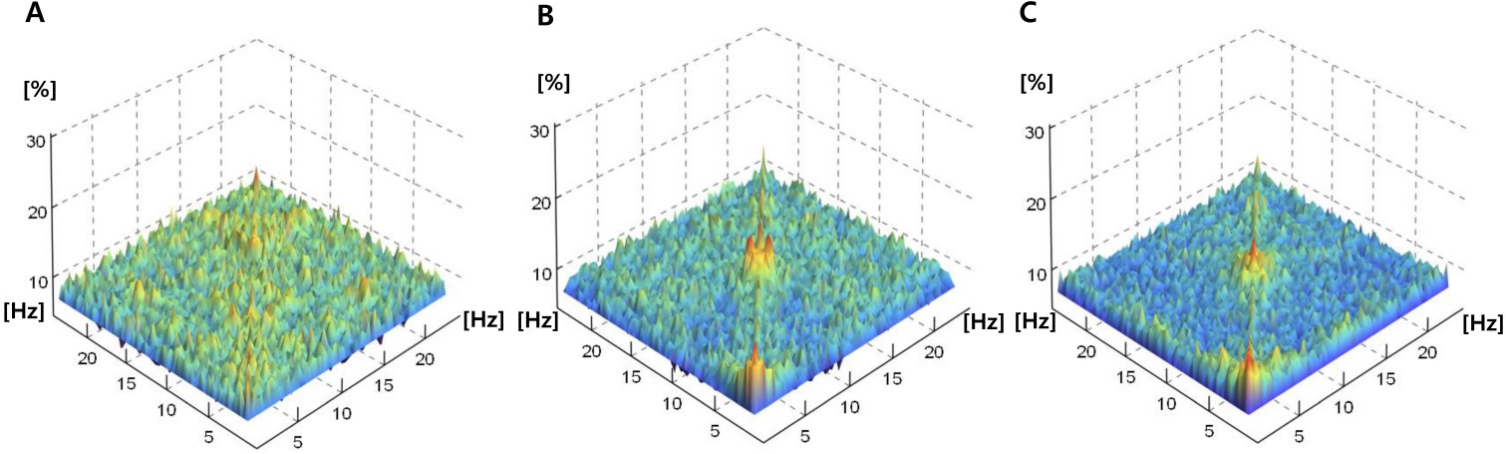
Averaged bicoherence spectra of all patients during light (**A**), moderate (**B**) and deep (**C**) dexmedetomidine sedation.

Fig. 2 shows the averaged bicoherence spectra of all patients at the three sedation levels. There were no noticeable increases in bicoherence during light sedation. However, there were two prominent bicoherences in areas around δ and α to spindle frequencies during moderate and deep sedation levels.

**Figure 3:**
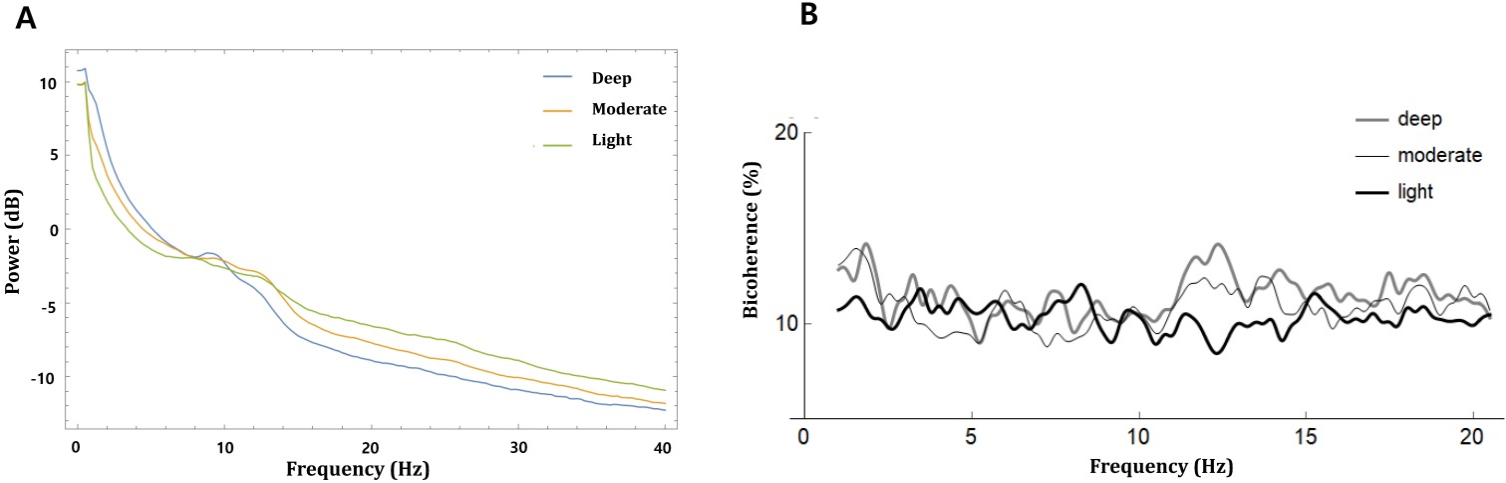
Power and bicoherence values along the diagonal lines of the bicoherence matrix under the three levels of sedation, shown as mean of all patients. **A,** Overlay of mean absolute power spectra of light (green), moderate (yellow), and deep sedation (blue); **B,** Mean bicoherence values along the diagonal lines of the bicoherence matrix of light (black), moderate (thin grey) and deep sedation (thick grey).

Fig. 3 shows the mean absolute power spectra and mean bicoherence values along the diagonal lines (f1 = f2) of the bicoherence matrix for all patients at the three sedation levels. In the power spectra, there were conspicuous peaks of around δ (<4 Hz) and α to spindle (9-15 Hz) during the three sedation levels. The bicoherence values along the diagonal lines of bicoherence matrix also showed two peaks in the δ and spindle frequencies that were almost consistent with the power spectrum. Therefore, we compared the δ (0.5-4 Hz) and α to spindle (9-15 Hz) peak bicoherences according to three sedation levels in the diagonal line.

**Figure 4:**
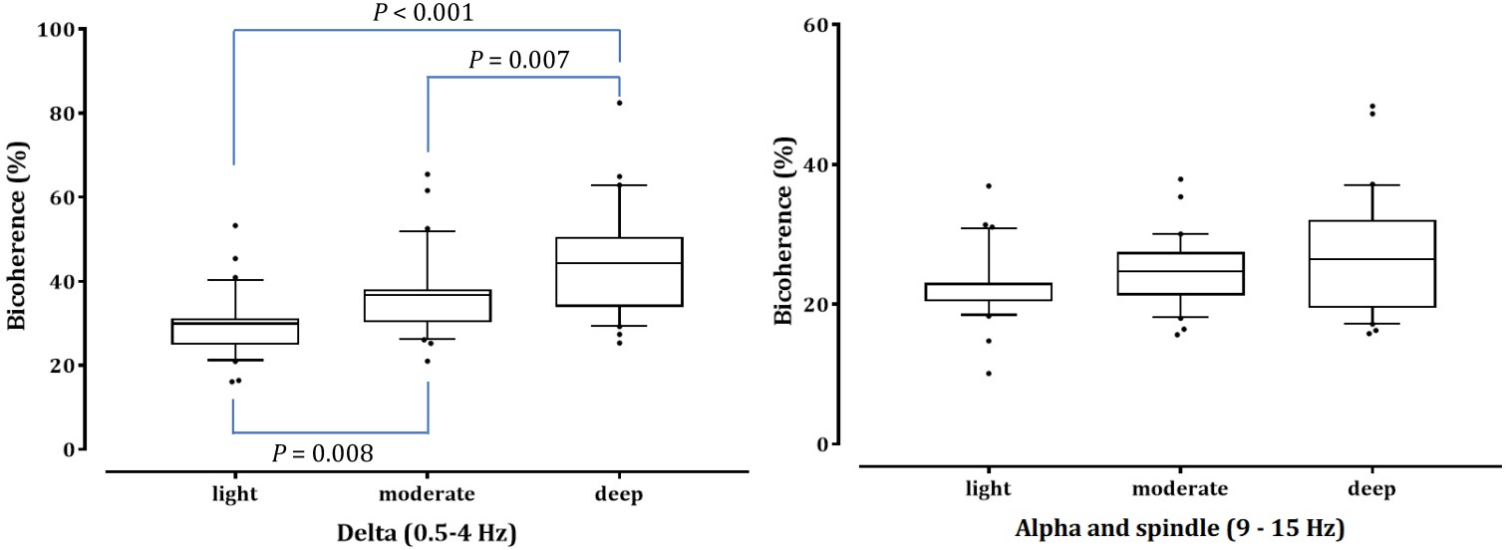
Peak bicoherence around diagonal lines in δ (0.5-4 Hz) and α-spindle (9-15 Hz) bands in all patients are respectively summarized in three sedation levels. The upper and lower limits of the box reflect the 75^th^ and 25^th^ percentiles of the sample, and the horizontal line inside each box indicates the median. The upper and lower notches reflect the 90^th^ and 10^th^ percentiles of the sample. Values below and above the notches are drawn as individual points. One-way ANOVA with repeated measures, followed by post hoc Bonferroni test (corrected *P* < 0.0167).

As shown in Fig. 4, the δ-peak bicoherence increased significantly with increasing sedation (*P* < 0.001). The δ-peak bicoherence was 29.93% ± 7.38%, 36.72% ± 9.70%, and 44.88% ± 12.90% (mean ± standard deviation) in light, moderate, and deep sedation, respectively (*P* = 0.008 and *P* < 0.001 in light sedation vs moderate and deep sedation, respectively; *P* = 0.007 in moderate sedation vs deep sedation). The α-spindle peak bicoherence was 22.92% ± 4.90%, 24.72% ± 4.96%, and 26.96% ± 8.42% in light, moderate, and deep sedation, respectively. However, there was no significant difference in the α-spindle peak bicoherence at the three-sedation level (*P* = 0.053). The δ peak bicoherence was significantly correlated with the depth of sedation (Spearman's ρ = 0.558, *P* <0.001)*,* but the α-spindle peak did not (Spearman's ρ = 0.190*, P* = 0.115).

## Discussion

In this study, we assessed EEG changes according to the depth of dexmedetomidine sedation in patients undergoing spinal anesthesia using power spectral and bicoherence analyses using EEG data from a single frontal electrode. BIS values, SEF95, and dexmedetomidine Ce were well-correlated with the depth of sedation, and* P_K_* values for the three measures were comparable. According to the gradual deepening of dexmedetomidine sedation, δ power increased and high-frequency powers decreased, but the increase of α and spindle powers was associated with light to moderate sedation. Dexmedetomidine caused bicoherence peaks in the δ and α-spindle frequency areas under moderate to deep sedation. Deepening of the dexmedetomidine sedation resulted in a significant increase in peak bicoherence of the δ area, although the peak bicoherence of the α-spindle area did not change.

Reliable monitors reflect physiological responses in their measurements. However, different anesthetics have different mechanisms and can lead to different EEG dynamics and anesthetic depth monitors' values, even for the same depth of anesthesia. For propofol anesthesia, the effect site concentration and level of sedation are known to be well correlated with BIS values 19; however, there are only few studies assessing how BIS reflects dexmedetomidine sedation in a clinical setting. Previous clinical studies mostly investigated the performance of BIS when dexmedetomidine was used as an adjuvant to other anesthetics 20, 21. In volunteer studies, the exact values were different depending on the studies; BIS was approximately 45-60 and 25-45 during moderate (MOAA/S 3) and deep (MOAA/S 1) sedation of dexmedetomidine, respectively 4, 22. BIS values were 10-20 lower than those in the same sedation level of propofol. Changes in BIS values according to the different sedation levels in this study were quite consistent with those of previous studies, but BIS values showed the greatest inter-individual variability during moderate sedation (MOAA/S 3). Our BIS findings included a wider range of sedation levels during spinal anesthesia, which can be used as a reference in other clinical settings.

The conventional EEG parameter, SEF95, has been an important EEG parameter for assessing the depth of anesthesia during surgery, similar to other EEG-derived parameters 23. SEF95 shows biphasic changes at low concentrations in some anesthetics, but usually decreases as the concentration of anesthetics increases 9, 24, 25. During propofol-remifentanil anesthesia, *P_K_* of SEF95 was similar to the *P_K_* of approximate entropy and BIS for moderate sedation and unconsciousness 24. In this study, as the depth of sedation increased, SEF95 decreased and showed a significant correlation; in addition, the *P_K_* value was comparable to that of BIS. However, in previous studies, the mean SEF95 was about 10-15 Hz at MOAA/S1 under propofol anesthesia 24, and about 10-12 Hz at a high concentration of sevoflurane anesthesia (1.4 minimal alveolar concentration [MAC] or 3 vol%) 9, 16, whereas in our study it was lower as 8.72 ± 1.52 during deep sedation. This seems to be due to dexmedetomidine, which has a different mechanism of action from these anesthetics. In addition, BIS and SEF95 showed a significant partial correlation, indicating that lower SEF95 values ​​would have contributed to lower BIS values than those of other anesthetics.

The results of this study revealed changes in relative frequency band power with the gradual deepening of dexmedetomidine sedation. Dexmedetomidine-induced sedation is known to have similar EEG changes in normal physiological stage 2 sleep that sleep spindles with maximal power at approximately 13 Hz appear during moderate sedation 5, 6. Therefore, we analyzed spectral band power subdivided into 6 frequencies, including the 12-15 Hz spindle. The results of this study are quite consistent with those of previous studies regarding absolute power changes 2, 5, 22, 26, 27. Previous studies conducted a spectral analysis of moderate and deep sedation with 0.7-1 μg/kg/h infusion of dexmedetomidine after loading dose and reported that δ, spindle, and θ power increased and β and γ power decreased during moderate and deep sedation compared to awake state 5, 22, 27. Sleigh et al. demonstrated that an increase in α (9-14 Hz) and δ (0.5-1.5 Hz) power was associated with mild to moderate and deep sedation, respectively, and that β (15-24 Hz) power decreased gradually with increasing plasma concentrations of dexmedetomidine; thus, β (15-24 Hz) power could predict the depth of sedation well 2. The continuous decrease in β and γ power in the findings of this study and previous studies may also explain the lower BIS and SEF95 values as compared to other anesthetics. In this study, relative α and spindle powers increased at light to moderate sedation compared to the awake state but decreased again at deep sedation. A study of spatiotemporal dynamics revealed that dexmedetomidine induced moderate sedation associated with increased α and spindle powers in frontal electrodes 27. Xi et al. also reported that spindle power across the cortex increased from wakefulness to moderate sedation and decreased again at deep sedation compared with moderate sedation 22. However, the other previous study reported that the α-spindle (8-14 Hz) power increased at moderate sedation (loss of responsiveness to auditory stimuli) compared to awake state but plateaued from moderate sedation to deep sedation (loss of consciousness) 26. Therefore, the decrease in relative α and spindle power at deep sedation may be due to the increase of the δ power, because total powers were comparable during different sedation levels in this study.

Although most previous studies were conducted in volunteers, we conducted dexmedetomidine sedation according to more subdivided levels in patients undergoing spinal anesthesia. We gradually increased the target effect site concentration of dexmedetomidine, which allowed us to investigate the effect of dexmedetomidine over a wider range of sedation levels, including light sedation. The animal experiment suggested that spinal anesthesia can indirectly depress cortical activity by decreasing excitability of reticulo-thalamo-cortical arousal mechanisms 28, but it has not been determined in clinical studies 29, 30. In the elderly, SEF90 decreased by the decrease of β power and increase of δ power 5 min after spinal anesthesia 29. However, in the middle-aged (51±14) patients, spinal anesthesia increased SEF95 by the increase of β power with decrease of δ power, similar to biphasic effect of low-dose anesthetics such as propofol and midazolam 30. In this study, spinal anesthesia seems to minimally affect the spectral power change of dexmedetomidine sedation because of the consistency of the result with previous volunteer studies. Nonetheless, it still needs to be validated in a larger group of patients undergoing other procedures to ensure applicability to a variety of clinical situations.

We also assessed how different dexmedetomidine sedation levels may affect the entire bicoherence spectrum in all pairs of frequencies. Bicoherence, the normalized parameter of bispectrum, is useful for extracting the distribution of frequencies and phase coupling data. Bicoherence analysis can detect a quadratic phase coupling and track changes in any reentry system including thalamocortical reverberating system 31. Strong phase coupling may imply sinusoidal component at two frequencies (f1 and f2) can have a common generator, or that the neural circuitry they drive may synthesize a dependent component at the modulation frequency of f1 + f2. Therefore, a phase-coupled peak is appeared in the corresponding bicoherence when a certain rhythm is formed in the reentry system. Dexmedetomidine induced spindles are thought to be generated by thalamocortical loop mechanism similar to propofol induced frontal alpha oscillations and sleep spindles 6, 7. Slow oscillations are also likely caused by decreased adrenergically-mediated excitatory inputs to basal forebrain, the intralaminar nucleus of the thalamus and the cortex 7. Therefore, an increase in quadratic phase coupling in the EEG during dexmedetomidine infusion may reflect the reverberating regulation between cortex and thalamus 10, 11. In this study, the mean bicoherence of all patients was less than 20% at all frequencies, even in the deep sedation level, which was lower than that in previous reports 12, 16. This may be because the underlying molecular and neuronal mechanisms of dexmedetomidine are different from other anesthetics although they act on the same underlying thalamocortical system 7. While propofol and inhalation anesthetics act as GABA receptor agonists, dexmedetomidine binds to presynaptic α_2_ adrenergic receptors on neurons projecting from the locus coeruleus (LC) 7. Next, the hyperpolarization of LC neurons decreases the norepinephrine release, resulting in the loss of inhibitory inputs from the LC to the preoptic area of the hypothalamus. Loss of inhibitory inputs from the LC activates inhibitory pathways from the preoptic area to the arousal center, resulting in sedation; therefore, dexmedetomidine may closely approximate NREM sleep 6, 7. Sleep and dexmedetomidine sedation induce different cortical and thalamocortical activity from propofol-induced unconsciousness 7. Dexmedetomidine-induced slow-wave oscillation and spindles are smaller in power and less coherent than those in propofol sedation, which may be due to brief interruptions in neuronal activity 5, 7. This lower level of disruption in neuronal activity under dexmedetomidine compared to propofol may result in smaller bicoherence. However, bicoherence analysis lacks information between different brain regions including thalamic regulation linking to the cortical area, because which is measured from a single electrode derived EEG. Therefore, future studies regarding the analysis of multiple channel EEG or local field potential in the thalamus are still required to reveal the complexity or diversity of communication between thalamocortical networks during dexmedetomidine sedation.

Increased bicoherence generally appears along the diagonal lines (f1 =f2) because a certain single frequency component includes both original and modulated reverberating subcomponents 9, 16, 32. It has been found that the bicoherence of δ-θ and α band along diagonal line is related to the concentrations of anesthetic agents 10, 12, 29. Propofol infusion is known to generate increased average bicoherence values around 2-6 and 10 Hz along diagonal lines of bicoherence matrix when the target Ce is 3.5 μg/mL compared to the awake state 12. During sevoflurane and isoflurane anesthesia, peak bicoherences of 2-6 Hz (δ-θ) and 7-13 Hz (α) gradually increase with increasing anesthetic concentration 10, 32. When the isoflurane and sevoflurane concentrations increase above 1.1% and 1.4%, respectively, the peak bicoherence of δ-θ maintains and peak bicoherences of α decrease. However, bicoherence changes in dexmedetomidine sedation have not yet been reported. In this study, all EEG bicoherence values were small and showed sporadic distribution during light sedation. When the dexmedetomidine sedation level was increased, two peaks around the δ and α-spindle frequencies appeared. In this study, we analyzed the changes of two peaks (0.5-4 Hz and 9-15 Hz) along the diagonal lines of the bicoherence matrix at different sedation levels. The increase in peak bicoherence of δ related to depth of sedation, and peak bicoherence of α-spindles increased slightly with increasing sedation, but the difference was not statistically significant. Peak bicoherence of α frequencies was approximately 20%-30% regardless of the depth of sedation and lower than those of previous studies 10, 12. This finding coincides well with previous studies that dexmedetomidine spindles are brief and episodic compared to highly coherent and continuous propofol α oscillations 5, 7. Changes in bicoherence were observed up to deep sedation in this study, and dexmedetomidine concentration could not be raised to a level that exceeded general anesthesia. Therefore, neither a plateau of δ peak bicoherence nor a decrease in α-spindle peak bicoherence was observed, unlike in previous studies on inhalation anesthetics 10, 32. However, in a clinical setting, dexmedetomidine is used at concentrations within the scope of our study and deep sedation usually requires ≤ MOAA/S 2. Pritchett et al. also reported that the bicoherence of δ (< 3.5 Hz) and δ-θ area (3.5-7.5 Hz) were more sensitive to anesthetic depth changes, and peak bicoherence showed stronger associations with anesthetic depth than average bicoherence 33. The findings of this study suggest that peak bicoherence of δ can be an indicator for evaluating the effect of dexmedetomidine sedation. Bicoherence spectra changes during dexmedetomidine sedation can also contribute to further development of DOA monitors that reflect the different EEG features of anesthetics.

In this study, cross-frequency coupling was not detected, which might be due to the Fourier-based method. A previous study performed phase-amplitude coupling (PAC) analysis between α and slow-δ bandwidths during dexmedetomidine infusion, but there were no differences in PAC strength between the different conscious or responsiveness states 26. Li et al. analyzed wavelet bicoherence during isoflurane anesthesia and revealed that the strength of cross-frequency coupling between α and slow δ waves changed according to the changes in isoflurane concentration 11. Therefore, further studies for cross-frequency coupling measured by bicoherence analysis are still needed in dexmedetomidine sedation.

This study has a few major limitations. First, we used EEG data from a single frontal electrode given that we implemented this study in surgical patients. The EEG analysis that was restricted to one or two different electrode combinations is compatible with the clinical setting, where frontal channels are easily accessed, but hardly can provide information on the actual signal sources or underlying networks. Although it has known that EEG reactivity for anesthetics is most prominent in the midfrontal derivations 26, 34, recent studies have revealed that the functional connectivity of different brain regions is changed related to anesthesia 5, 35. Kallionpaa et al. suggested that prefrontal-frontal EEG-based connectivity could discriminated the states at the different dexmedetomidine concentrations 35. If more EEG data have been recorded in different brain regions, it would have been possible to assess the spatiotemporal dynamics and connectivity changes of dexmedetomidine sedation in surgical patients. Second, the MOAA/S scale can be a relatively subjective finding. Although the MOAA/S scale is commonly used in a clinical setting, it can suffer from the 'observer effect' which is open to interpretation. Therefore, observer bias may have impacted the result of the present study. Third, the bicoherence spectra of the awake state were not included in the analysis. The pattern of bicoherence spectrum in the awake state is known to vary depending on the physiologic status of the subjects 10. Finally, as this study was not a confirmatory study to test a hypothesis, but sought to explore an underlying mechanism, it was not possible to predefine the effect size. Therefore, the sample size was not calculated. As such, it remains possible that our findings may be underpowered.

## Conclusions

In this study, we demonstrated the hierarchal EEG changes of dexmedetomidine sedation in patients undergoing spinal anesthesia using the EEG data from a single frontal electrode. BIS, SEF95, and dexmedetomidine Ce were well-correlated with the depth of sedation and could similarly predict sedation levels but the interindividual variability of BIS was highest during moderate sedation. Since the changes in the relative band power according to sedation level were consistent with those of volunteer studies, spinal anesthesia did not seem to have a significant effect on the power spectrum change. The bicoherence peaks of δ and α-spindle regions emerged during moderate and deep sedation, and the δ bicoherence peak was suggested to be an indicator reflective of sedation levels of dexmedetomidine.

## Figures and Tables

**Table 1:** Demographic and perioperative data of the patients

Variables	Number or Median (Minimum - Maximum)
Sex (male/female)	12/18
Age (years)	54 (20-70)
ASA physical status (1/2)	13/17
Body weight (kg)	64 (51-86)
Height (cm)	164 (140-180)
BMI (kg/m^2^)	24.33 (20.08-29.90)
Bupivacaine dose (mg)	11.5 (10-14)
Sensory block level	T10 (T3-T12)
Duration of dexmedetomidine infusion (min)	73.5 (46-141)
Total dose of dexmedetomidine infused (μg)	156.7 (91.7-267)
**Type of surgery**	
Arthroscopic knee surgery	18
Total knee replacement	2
O/R and I/F of ankle	6
Etc.	4
**Side effects (n[%])**	
Hypertension	15 (50%)
Bradycardia	11 (36.67%)

ASA: American Society of Anesthesiologists, BMI: body mass index, T: thoracic dermatome, number or median (range) O/R and I/F: open reduction and internal fixation.

**Table 2:** Bispectral index, 95% spectral edge frequency and effect site concentration of dexmedetomidine at different sedation levels

	MOAA/S 5 (baseline)	MOAA/S 4	MOAA/S 3	MOAA/S 2	MOAA/S 1	*P_K_* (SD, 95% CI)
BIS	93.98±3.38 (3.60)	71.50±7.42 (10.38)	61.15±10.46 (17.11)	48.11±6.81 (14.16)	40.23±6.62 (16.46)	0.847 (0.002, 0.842-0.853)
SEF95	22.75±4.18 (18.37)	17.83±3.29 (18.45)	14.19±3.15 (22.20)	10.62±1.63 (15.34)	8.72±1.52 (17.43)	0.841 (0.003, 0.836-0.846)
Dex Ce	0	0.82 ± 0.09	0.97 ± 0.15	1.18 ± 0.19	1.39 ± 0.13	0.844 (0.003, 0.839-0.849)

Data are presented as mean ± standard deviation (coefficient of variation). Coefficient of variation = standard deviation/mean × 100%. MOAA/S = Modified Observer's Assessment of Alertness/Sedation scale. *P_K_* = Prediction probability, BIS = bispectral index, SEF95 = 95% spectral edge frequency, Dex Ce = effect-site concentration of dexmedetomidine.
